# Characterization of cannabis withdrawal symptoms and serum levels of neurotransmitters among cannabis-dependent smokers during sustained abstinence within a controlled residential environment

**DOI:** 10.1192/j.eurpsy.2023.1195

**Published:** 2023-07-19

**Authors:** R. Sharma, S. K. Tikka, A. R. Bhute, P. Dhamija, B. K. Bastia

**Affiliations:** 1 Forensic Medicine and Toxicology, AIIMS, Rishikesh, Uttarakhand; 2 Psychiatry, AIIMS, Bibinagar, Telangana; 3 Pharmacology, AIIMS, Rishikesh, India

## Abstract

**Introduction:**

Cannabis (aka marijuana) is the most frequently consumed illicit substance worldwide, and a subset of frequent cannabis smokers (up to 30%) develop dependence. A less well-known consequence of cannabis dependence is withdrawal syndrome, characterized by a time-dependent constellation of symptoms (Lafaye et al. Dialogues Clin Neurosci 2017;19(3), 309-316).

**Objectives:**

This study aims to prospectively assess the course of cannabis withdrawal symptoms within a controlled inpatient detoxification setting and to correlate the severity of withdrawal symptoms with the serum levels of neurotransmitters (NT).

**Methods:**

N=45 treatment-seeking chronic cannabis dependents (assessed by ICD-10) were enrolled, and their withdrawal symptoms were assessed prospectively from admission (Day-0) to 28 days using Marijuana withdrawal checklist (MWC). Sociodemographic characteristics and self-reported drug use histories were reported. Serum levels of dopamine, serotonin, norepinephrine, epinephrine, and cortisol were measured. Cannabis abstinence symptoms were assessed daily using MWC for 4 weeks, and serum neurotransmitter levels were analyzed at admission (Day 0), 7, 14, 21, and 28. Comparison between groups was done using Friedman’s test. Correlation between NT level and MWC scores was performed using linear regression spearman correlation analysis

**Results:**

The follow-up NT levels from Day 0 to 28 showed a significant (p<0.05) decrease in serotonin and dopamine, whereas epinephrine levels showed a significant increase (Fig 1) with the course of withdrawal. Withdrawal symptoms like decreased appetite, sweating, and craving were significantly and positively correlated with serotonin, dopamine, and epinephrine NT levels (Fig 2).

**Image:**

**Figure:**
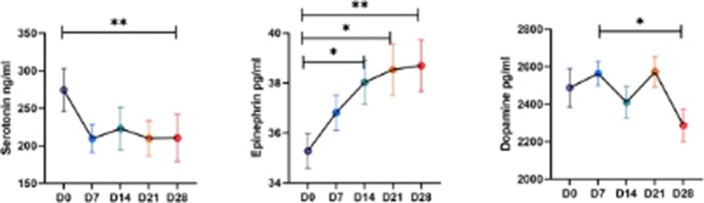

**Image 2:**

**Figure:**
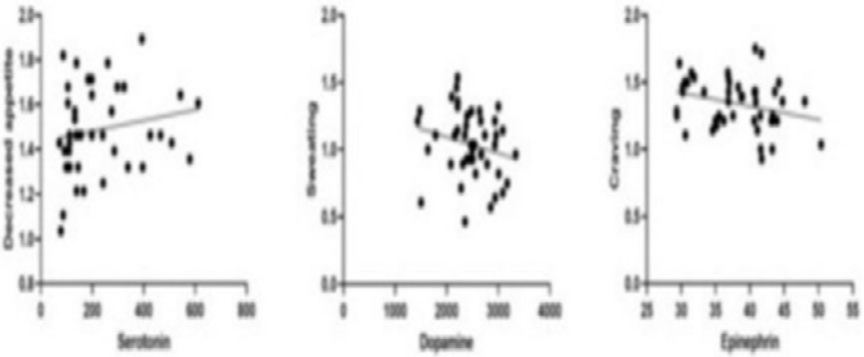

**Conclusions:**

Findings support the presence of clinically significant cannabis withdrawal symptoms with NT levels in subjects with cannabis dependence seeking substance abuse treatment. The data of this study determine the relationship between observed withdrawal symptoms and changes in brain chemistry and evaluate its possible utility as a predictor of relapse.

**Disclosure of Interest:**

None Declared

